# The Remedies of Nature

**Published:** 1889-06

**Authors:** 


### "THE REMEDIES OF NATURE."

In a series of papers on "The Remedies of Nature," an eminent English physician comments upon past and present medical treatment ; and though, presumably, it is the intention of these papers to convey important and timely information to the medical faculty, they furnish at the same time a hint to the patient at large which, if he be wise, he will hasten to avail himself of. Perhaps it is the author's intention that he should, for at one moment he seems to drop his voice to a whisper while admonishing the fraternity that they must stop dosing and drugging, and at another talks in stentorian tones over their heads, warning the public to look to nature rather than to art for relief from all the minor ailments to which humanity is heir. It may, perhaps, be a disappointment to them who have come to lean upon their medical adviser for advice, and rely upon him for health, to learn that the effects of fresh air are more potent and enduring than artificial stimuli, and exercise more to be depended on than jalap, attenuations of aconite and belladonna, or even bread pills. He inveighs against the practice, now unhappily prevalent, of attacking the effects or outward signs of a disease instead of the cause or seat of the malady—a practice which sometimes proceeds from ignorance, though it is often adapted to allay the fears of the patient.

"A swelling suddenly appears on a man's knee, whereat," says the author, "he flies in alarm to his physician. The latter sets himself diligently to work to remove the swelling, and to the joy of his patient, succeeds."

This, he says, is like stopping the alarm bells which tell us that a fire is broken out. We should be attending to the fire and let the bells ring. The swelling on the man's knee might not, it seems, be a disorder in itself, but only the outward expression of a real trouble existing within —a warning given by nature, and perhaps an outlet, which, if encouraged rather than restrained, might do much to alleviate the disorder of which it is the expression.

He does not believe in giving drugs and medicines of any kind—he does not refer to simples—save in extreme cases, because their use puts an extra tax on the strength of the patient, who, after recovering from the original malady, must also recover from the effects of the foreign substances that have been taken into the body or injected under the skin.

A man, woman, or child who will take a fair proportion of fresh air and exercise daily will not fail to be benefited in health. The effects of fresh air and exercise, when taken continually, would seem exaggerated if set down here—let those who may be interested inquire at the nearest gymnasium.

McClellan, the boxing master at Wood's Gymnasium, in New York City, said recently to the writer: "The doctors couldn't do anything for Mr. —— (once a confirmed invalid). I took hold of him, made him box with me; a very little at first, increasing the amount of the exercise as the weeks went by, until now he is quite recovered, goes to his office every day, and walks up and down town in all weathers. He eats well and sleeps well—it all came along of the boxing."

This man used to be surrounded by bottles containing medicine, like an apothecary's clerk in a compounding room. He took something out of one bottle when he got out of bed in the morning, and helped himself from others before and after each meal. The more medicine he took the feebler he appeared. One malady seemed only to pave the way for another, ache followed ache, what brought relief to one ailment added to the intensity of another, and he soon found that thus to seek for health by way of the materia medica was, like the first inhabitants of Arcadia, to chase the sun, which, when they had reached the hill on which it seemed to rest, was still beheld at the same distance from them.

It is not intended to cast a reflection upon those estimable and skilful physicians to be found to-day in almost every community, who are quick to recognize symptoms, adroit in lessening pain, and with whom the saving of life is a common incident. But many, perhaps it is safe to say most, physicians, do little to encourage the ailing to rely upon their own exertions for relief, rather than upon medicines, which at best can afford but a temporary respite from suffering and disease.—*Scientific American.*

